# Investigating the impact of acceptance and commitment therapy for mental healthcare professionals: the effect on patients´ self-stigmatization, a pilot study

**DOI:** 10.3389/fpsyt.2024.1390949

**Published:** 2025-01-07

**Authors:** Kim L. Helmus, Marilon van Doorn, Mariken B. de Koning, Inez Myin-Germeys, Frederike N. Schirmbeck, Therese A. M. J. van Amelsvoort, Dorien H. Nieman, Monique W. M. Jaspers, Arne Popma, Lieuwe de Haan

**Affiliations:** 1 Research Mental Health, Amsterdam Medical Centre, Amsterdam, Netherlands; 2 Neurology and Psychiatry Department, Amsterdam Universitair Medisch Centrum, Amsterdam, Netherlands; 3 Severe Mental Health Care Department (Mentrum), Arkin, Amsterdam, Netherlands; 4 Research Department, Arkin, Amsterdam, Netherlands; 5 Faculty of Medicine, Katholieke Universiteit Leuven, Kortrijk, Belgium; 6 Faculty Health, Medicine and Life Sciences, Maastricht University Medical Centre, Maastricht, Netherlands; 7 Department of Medical Informatics, Amsterdam Public Health Institute, Amsterdam, Netherlands

**Keywords:** self-stigmatization, acceptance and commitment therapy (act), mental healthcare, professionals, stigma & awareness

## Abstract

**Background:**

A significant proportion of mental health care professionals (MHCPs) hold stigmatizing attitudes about their patients. When patients perceive and internalize these beliefs, self-stigmatization can increase. Acceptance and Commitment Therapy (ACT) may decrease stigmatizing attitudes by changing the ‘us’ versus ‘them’ thinking into continuum beliefs. In the present study MHCPs were given an ACT-based training, aiming to decrease stigmatization, hypothesizing that self-stigmatization of their patients will subsequently decrease.

**Methods:**

An RCT with a 2 (pre-test/post-test) x 2 (no training/training) design was conducted. A total of 41 MHCPs participated, 20 were randomized to the experimental and 21 to the control condition respectively. The MHCPs in the experimental condition received an ACT-based training, MHCPs in the control condition received no training. From every MHCP, one of their patients participated in the pre- and post-measurement. As the primary outcome, patients’ awareness, agreement, application and hurt-self, was measured using the Self Stigma of Mental Illness Scale - Short Form (SSMIS-SF), before and after the MHCPs’ ACT-based training.

**Results:**

Significant group x time interaction effects were found for ‘application’ (internalization of mental illness stereotypes) in patients after the ACT-based training of their MHCP: *F* (1,39) = 9.33, p < 0.01, η_p_^2^ = .85. On the contrary, no effect was found on the subscales ‘awareness’, ‘agreement’ and ‘hurt-self’.

**Conclusion:**

Preliminary results suggest that a brief ACT training for MHCP might heighten their awareness and contribute to reduction of their stigmatizing attitudes and behavior, leading to less application of self-stigmatizing beliefs in their patients.

## Introduction

Previously, severe mental illness was predominantly regarded as chronic and deteriorating. However, over recent years there has been a shift towards more recovery-oriented perspectives and approaches (1, 2) Numerous studies demonstrate that many individuals with severe mental illness achieve recovery in terms of symptomatology, (social) functioning or/and personal growth (3–5). Despite the increasing acceptance and comprehension of these recovery-oriented approaches, the implementation and delivery of services continues to pose significant challenges (2, 6).

The prevalence of stigmatization towards patients with severe mental health problems contributes to the challenges in implementing recovery-oriented approaches (7). Even though mental healthcare professionals (MHCPs) themselves do not always recognize having stigmatizing attitudes (8), stigmatization within mental healthcare remains common (9, 10). Disturbingly, over twenty percent of reported instances of stigmatization are attributed to encounters with MHCPs (10) even though attitudes of MHCPs seem overall more positive compared to the general public (11). One of the underlying causes can be a binary perspective held by some MHCPs, where mental health and mental illness are perceived as fundamentally distinct, leading to stereotypes and a separation of ‘us’ versus ‘them’; seeing ‘them’ as different from their own ‘normal’ experiences and behavior. Stereotypes and beliefs can be implicit (i.e., beliefs that reside outside of conscious control and/or awareness; associations that one would not explicitly endorse or reveal) or explicit (beliefs that are self-reported and occur within conscious control and/or awareness). There is an increasing understanding that explicit measures may underestimate levels of stigmatization (12, 13). Further, explicit and implicit measures may differentially predict behavioral outcomes due to operating through reflective (e.g., basing decisions on knowledge about facts and values) versus impulsive (e.g., basing decisions on associative links and motivational orientations) systems (14), or based on whether outcomes are controllable or spontaneous (15). Both implicit and explicit stereotypes and beliefs are associated with labeling, and can result in the marginalization and discrimination of a specific group. This categorical distinction plays a significant role in the stigmatization process (16). Stigmatization of individuals or groups with mental health problems involves perceiving, experiencing, or labeling mental conditions or issues as deviant or negative, leading to the detrimental consequences of discrimination, prejudice, exclusion, stereotyping, and status loss (17).

Potential consequences of stigmatization are a decrease in life satisfaction, lesser likelihood of help seeking behavior, less employment chances, less housing opportunities, fewer social opportunities and received healthcare is of inferior quality (18–23). Stigmatization does not affect all individuals with mental health problems in the same way. Some respond with indifference towards stigmatizing behavior and others show high levels of psychological resilience, which may even fuel their motivation to challenge negative beliefs (24). However, the majority of individuals tend to endorse and thereby suffer from stigmatizing attitudes (25). When someone considers stigmatizing beliefs to be true, these beliefs can be internalized and cause self-stigmatization. Self-stigmatization is the process in which an individual thinks that he or she is socially less acceptable and/or valuable, it involves identification with a stigmatized group, and the internalization of the negative attributes and stereotypes associated with this group (26). Self-stigmatization is prevalent and is related to demoralization, reduced physical and/or mental health and a diminished self-efficiency, self-esteem and quality of life (27) (25, 28) (29–31).

Several meta-analyses on the effectiveness of interventions to diminish stigmatization (16, 32, 33) and self-stigmatization (24, 31, 34, 35) have been published. These include psycho-education programs, trainings, individual- and group sessions. The results vary substantially; some studies show no effects, while others report high effectiveness in reducing stigma. One of the challenges in providing stigma-reduction trainings is that people often resist acknowledging or recognizing stigmatizing beliefs in themselves because this threatens their self-image. This can cause social desirability and consequently unreliable outcomes on stigma measurements (36, 37). Recently there has been a shift towards recommending promoting continuum beliefs (e.g. understanding that mental health problems can be seen on a continuum from mild to severe; to some degree all humans suffer from them) rather than explicitly targeting the reduction of stigmatizing attitudes (16, 37). Additionally, an ethical question arose while reviewing self-stigma intervention studies: shouldn’t the first step be to offer interventions to mental health care workers (MHCWs) before addressing self-stigmatization in patients? This is important because, for many patients, self-stigma often begins within mental health care and is driven by labelling and stigmatization. It seems reasonable to tackle self-stigma by focusing on stigma reduction among mental health care workers (38). Given that categorical beliefs play a pivotal role in the stigma process, continuum beliefs can be regarded as a countermeasure against stigmatization (16). We conducted a pilot study to understand the effects of an intervention enhancing continuum beliefs, based on acceptance and commitment therapy (ACT), offered to MHCPs to reduce self-stigmatization in their patients. A continuum concept of mental health and mental illness assumes one dimension from severe psychiatric symptoms to subclinical, light, or non-existent symptoms. Since every person is likely experiencing symptoms of mental illness at some points during their life, a person with severe mental illness might be seen as someone with similar, but with more severe experiences, thus remaining ‘someone like us’ (16). In ACT, mental health problems are explained using continuum beliefs, understanding that we all experience emotional difficulties to some extent and in everyone the intensity of the struggle fluctuates over time. Furthermore, ACT enhances psychological flexibility, which is defined as the ability to maintain a gentle and kind awareness of thoughts, emotions, and the present moment, and take action, based on personal values and goals. Psychological inflexibility is found to be positively related to stigmatization (39, 40). ACT interventions are promising in the reduction of (self)stigma of mental health problems (38, 39, 41). The current study focusses on whether self-stigmatization in patients decreases after their MHCP received ACT, with a focus on enhancing continuum beliefs. It is hypothesized that after the intervention for their MCHPs, patients’ agreement with, beliefs about and application of common mental health stereotypes as well as the ‘hurt-self’ (self-esteem or self-efficacy as a consequence of internalizing these stereotypes) decreases compared to the control group.

## Methods

### Participants and design

**Figure 1:**
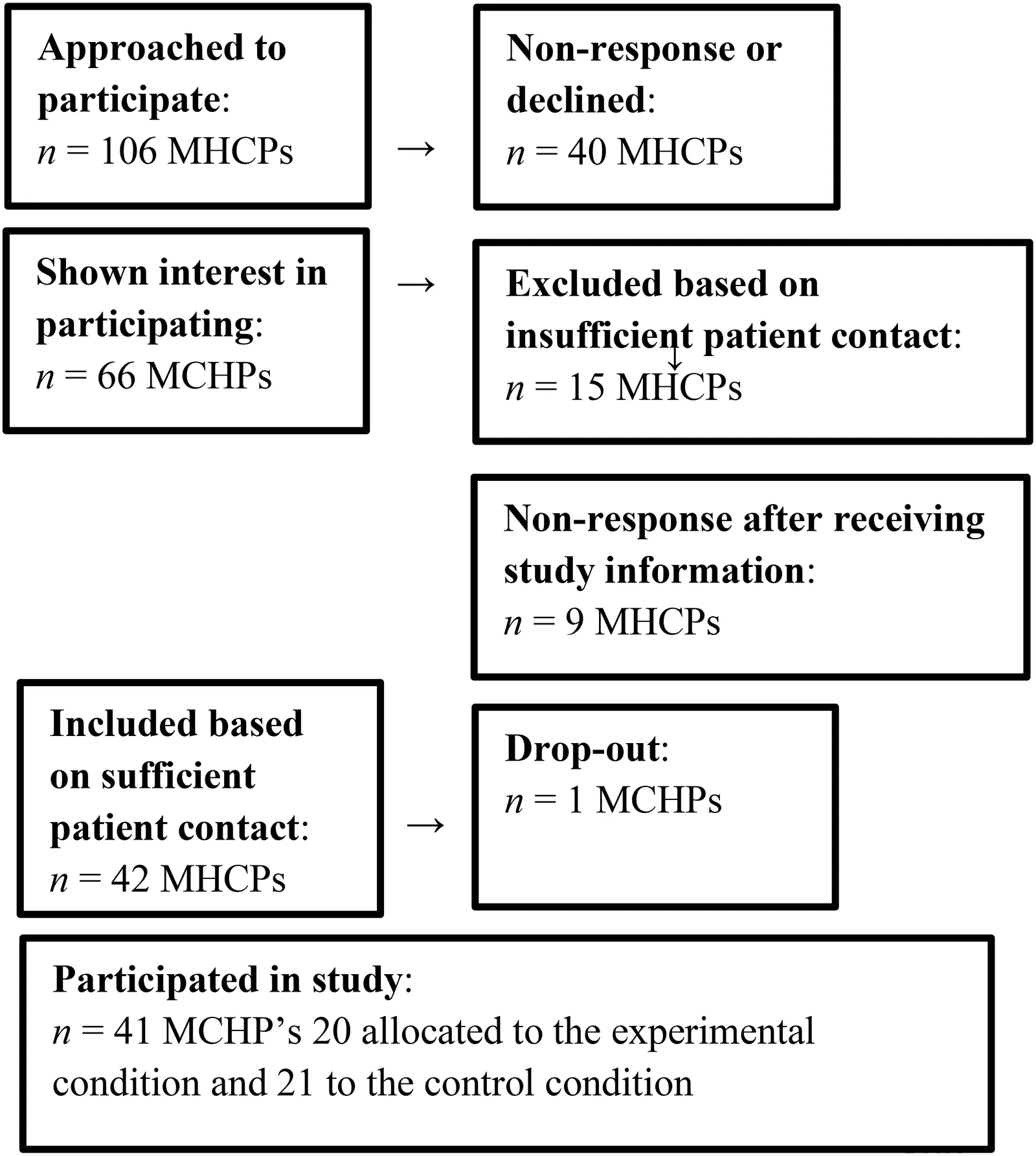
Flowchart MHCPs.

A total of 106 MHCPs from the mental health organization Arkin - Mentrum Amsterdam in the Netherlands were randomly selected out of a list of employees (**Figure 1**: Flowchart MHCPs). MHCPs worked at different departments and teams for treatment of patients with severe mental illness (SMI) at clinics or outreaching teams; e.g. (Functional) Assertive Community Treatment teams. They were invited to participate in the study and informed via email which contained an informed consent form and a demographic questionnaire. The aim was to obtain a diverse sample (social psychiatric nurses, psychiatrists, psychologists, case managers and job coaches all working at teams for treatment of people with severe psychiatric disorders). 42 out of the 106 MHCPs met the inclusion criteria and agreed to participate, one dropped out at the beginning of the study due to work appointments. The inclusion criterion for the MHCP was that during the study they would be able to arrange face to face contact with the participating patient weekly. For MHCP characteristics see **Table 1**. The number of participants needed for the study was determined by means of G*power analysis for which an alpha level of.05 and a power of.80 were used (42). Considering the effects from previous research, a medium effect size of.25 was expected, resulting in 28 participants for testing a time*condition interaction (24, 41).

**Table 1:** Demographic characteristics of participants, N = 41.

Clinicians					
Age	Gender	Education	Job	Working	Encounter
40.0 (1.81)	32% Men 68% Women	2% Senior general secondary education 12% Intermediate vocational education 85% College	12% Society worker 39% Psychiatric nurse 7% Community worker 2% Patients confidant 7% Psychologist 15% Job coach 15% Probation officer	11.6 years (1.42 SD)	54% Others 5% Self 34% Both 7% None
Patients					
Age	Gender	Education	Living situation	Years MP	Marital status
37.6 (2.19)	78% Men 22% Women	22% Primary school 17% Lower general secondary education 15% Intermediate vocational education 27% College	46% Independent living 2% Student flat 20% With parents 20% Sheltered housing 7% Admitted in mental health hospital 2% Different	17.4 (2.57SD)	95% Unmarried 5% Divorced

Means (SD) of clinicians age, amount of years working in mental health care (Working), percentages of gender, education, job, and having had the encounter of mental health problems in personal life with self and/or others (Encounter). Means (SD) of patients age and the number of years having mental health problems (Years MP), gender, education, living situation and marital status.

The study was approved by the Faculty Ethics Test Board of the University of Utrecht (FETC16-132). The design was an RCT with a pre-test and post-test measurement. Patients received treatment for severe mental illness (SMI) at clinics or outreaching teams; e.g. (Functional) Assertive Community Treatment teams. SMI is defined as a mental, behavioral, or emotional disorder resulting in serious functional impairment, which substantially interferes with or limits one or more major life activities (43). Most patients received several diagnoses within the spectrum of psychotic disorders, affective disorders, addiction and/or personality disorders accompanied by severe social and societal limitations, mostly persisting for several years. A coupled participant pool was used; every MHCP had to ask one of their patients with whom they were having weekly face-to-face sessions. This was done by telling them to invite the first patient they would meet in the coming week after the contact with the research assistant. 20 MHCPs were randomly assigned to the experimental condition and 21 MHCPs to the control condition, the MHCPs in the control condition were offered the intervention after the study. There were no exclusion criteria for the patients; all were allowed to participate regardless of age, how long they had been receiving treatment (this varied from being in their first week till many years) diagnosis, background, severity of problems or other characteristics (for characteristics of patients see **Table 1**).

### Intervention

**Figure 2:**
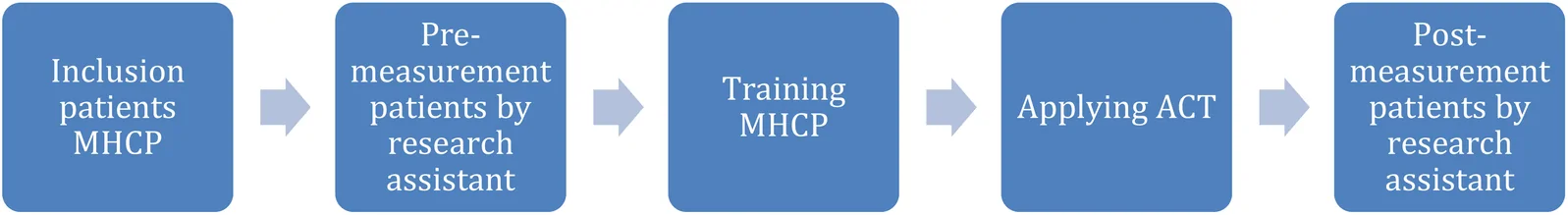
Study process description.

The training offered ACT based tools to work in a ‘destigmatizing’ way; stimulating disclosure, psychoeducation about continuum beliefs and psychological flexibility around stigmatizing beliefs (44, 45). The training took half a day (four hours) and combined a theoretical presentation and practical application of ACT to enhance acceptance, cognitive defusion interventions, defining values, connecting to and allowing the present moment and committed action. The theoretical framework of ACT that was used in this training had the aim to understand and create flexibility around stigmatizing ‘us vs them’ beliefs (‘I am healthy, you are sick’ or ‘Mental health problems are not acceptable’) and to practice continuum beliefs holding an inclusive and accepting perspective. During the training, the MHCPs were motivated to apply the ACT attitude and theory in the sessions with their patients. The reason for short duration of the training was that MHCP did not have more time in their schedule for in depth education. There are some effective interventions of ACT where short trainings or therapies were offered from 2,5 hours (46, 47) to 5 hours (47). The sessions the MHCPs had with their patient before and after the intervention took between 45 minutes to 60 minutes; the exact content of the sessions depended on the nature of the relation between the MHCP and the patient and the kind of sessions/treatment patients received and where they were in their treatment. All MHCPs’ sessions included at least a discussion of how the patient fared and whether there had been any difficulties or positive events and a reflecting on and/or evaluation of the treatment plan. The time of MHCP and patients knowing each other varied between having their first session together and knowing each other already for many months. The MHCPs in the experimental condition received the four-hour training in groups of ten, given by a licensed healthcare psychologist certified and experienced in ACT; Examples of exercises to practice with their patients after the intervention was offered were: psychoeducation about continuum beliefs of mental health disorders, sharing one’s own experiences with emotional difficulties, empathy and compassion enhancing experiential exercises and cognitive interventions on judgmental thoughts. The MHCPs participated in the ACT-training after the first measurement of their patient and had to apply ACT in contact with their patient within two weeks after the training. The post-measurement of the patients was done within one to two weeks after (see also **Figure 2** for the study process description).

### Materials

A validated Dutch version of the Self Stigma of Mental Illness Scale - Short Form [SSMIS-SF (32)] was used which consists of 20 items scaled on a 9-point Likert scale anchored at 0 (I strongly disagree) to 9 (I strongly agree). The psychometric qualities of the SSMIS-SF are good: the internal consistency lies between a = .72 and a = .91 and the test-retest reliability ranges from.68 to.82. Moreover, the content validity and construct validity are considered good (32, 48). The questionnaire measures the process of self-stigmatization, consisting of four stages and subsequently subscales, namely *Awareness*, which measures the knowledge of the common stereotypes in society (this sub-scale was adjusted for the current study to measure perceived stigmatization of a patient’s MHCP, item example: ‘I think that my MHCP thinks that most persons with mental health problems will not recover or get better’); *Agreement*, which measures the agreement with common stereotypes about individuals with mental health problems (item example: ‘I think that most persons with mental health problems are dangerous’); *Application*, which measures the internalization of common stereotypes about mental health problems (item example: ‘Because I have mental health problems, I am unpredictable’) and *Hurt Self*, which measures self-esteem or self-efficacy as a consequence of internalizing these stereotypes (item example: ‘I currently respect myself less because I am unable to take care of myself’). The SSMIS-SF consists of four valid and reliable sub-scales measuring different aspects of (self)-stigmatization; the total score should not be used as an aggregated measure. Therefore, scales have to be interpreted separately. The subscales have been found to be reliable with Cronbach’s alpha coefficients of .72 for Agreement, .81 for Application, .88 for Hurts Self, and .91 for Awareness (27, 49). Also, a demographic questionnaire for patients and a demographic questionnaire for MHCPs, including an item regarding personal experience with mental health problems was used.

### Statistical analysis

The data were analyzed using a 2 (pre-test/post-test) x 2 (no training/training) repeated measures design multivariate analysis of variance (MANOVA). An alpha of .05 was used and two-tailed tests were reported. In order to measure the effectiveness of the training, scores of patients in the control group versus scores of patients in the experimental condition were measured by means of interaction effect of time and condition.

## Results

No significant differences were found between characteristics of patients and MHCPs in both conditions (the age of MHCPs, the amount of years working in mental health care, the age of patients, the amount of years of patients having mental health problems, gender, having mental health problems in loved ones or personal mental health problems in MHCPs, the function of MHCPs, gender of patients, marital status of patients, and living situation of patients).

No significant differences between conditions on the SSMIS-SF were found, t(39) = -0.43, p = .67, indicating that pre-test self-stigmatization scores were not different over conditions (see **Table 2** for descriptive statistics).

**Table 2:** Descriptive Statistics for SSMIS-SF Scores.

Scale	Pretest M (SD)	Pretest N	Pretest Min	Pretest Max	Posttest M (SD)	Posttest N	Posttest Min	Posttest Max
Agree	18.43 (8.57)	21	5	45	17.43 (7.78)	21	5	37
Apply	13.48 (6.74)	21	5	28	12.10 (5.55)	21	5	25
Aware	19.52 (11.92)	21	5	42	17.86 (10.10)	21	5	38
Hurts Self	10.86 (6.24)	21	5	32	11.43 (6.84)	21	5	29

Considering Application, no significant differences between conditions on pre-test scores were found, t(39) = -1.71, p = .10, indicating that pre-test Application scores were not different over conditions. No significant differences between conditions on Agreement, t(39) = 0.74, p = .46, Awareness, t(1, 33.52) = 1.29, p = .21, and Hurt-self, t(1, 33.11) = -1.98, p = .06, were found, indicating that pre-test scores were not different over conditions.

No effect of time was found on the subscale Awareness, *F*(1,39) = .04, p = .85 and no effect of condition was found for Awareness, F(1,39) = .85, p = .36, also no significant interaction effects were found *F*(1,39) = 2.02, p = .16 suggesting that the ACT-training had no effect on the ‘Awareness’ subscale, compared to the control condition.

**Figure 3:**
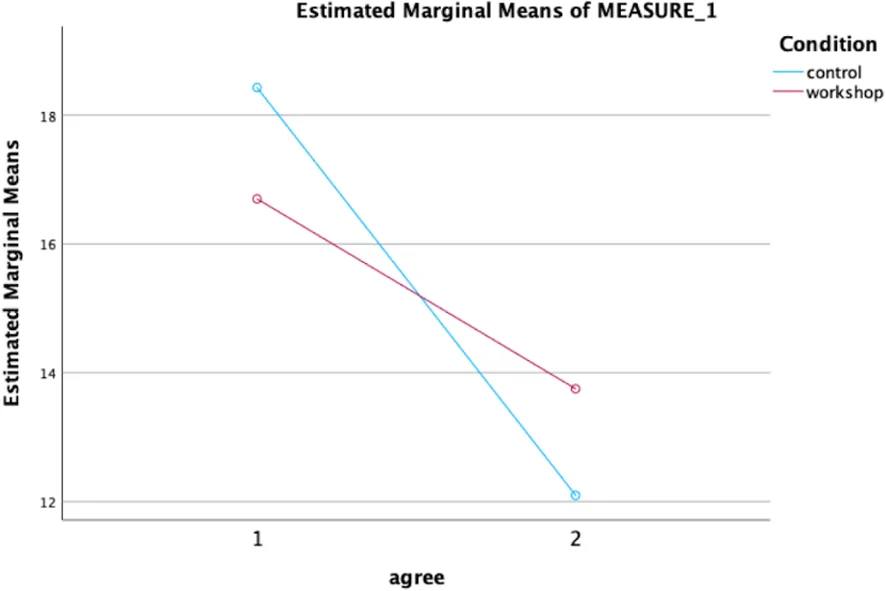
Pre- and post-test means (SD) of Agreement-scores per condition.

For the subscale Agreement, a significant effect of time was found (**Figure 3**), *F*(1,39) = 9.86, p <.01 η_p_^2^ = .87, meaning that over time Agreement scores decreased in both conditions. No significant effect of condition was found for Agreement, *F*(1,39) = .00, p = .98, and no significant interaction effect was found, *F*(1,39) = 1.31, p =.26. suggesting that the ACT-training had no effect on the ‘Agreement ‘ subscale, compared to the control condition.

**Figure 4:**
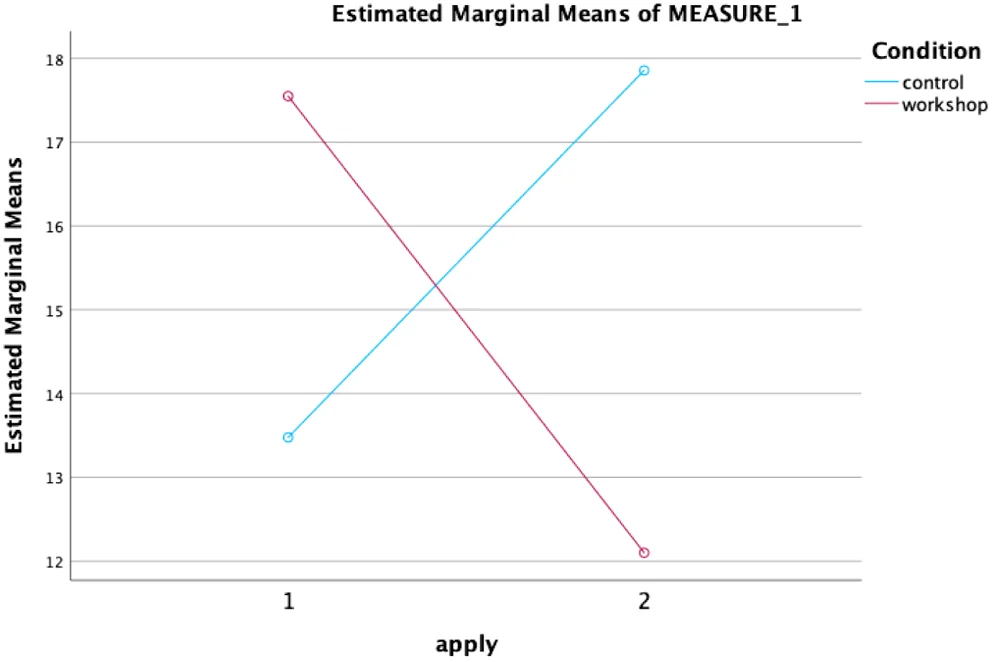
Pre- and post-test means (SD) of Application-scores per condition.

**Figure 5:**
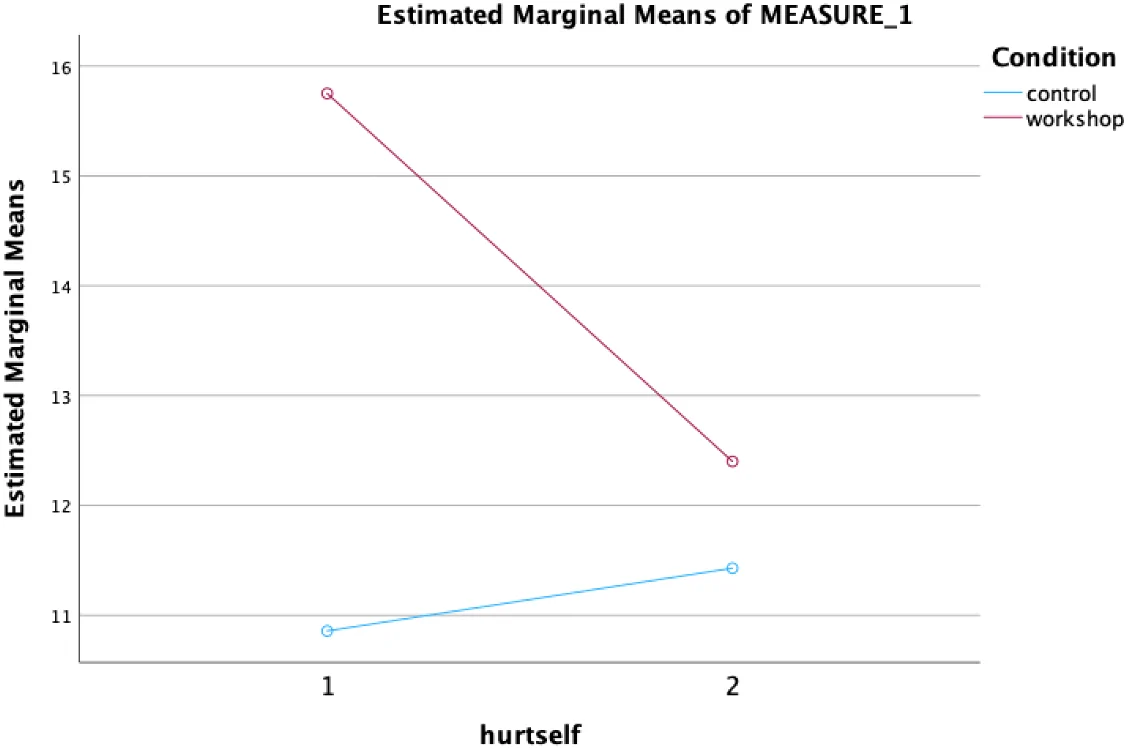
Pre- and post-test means of Hurt-self scores per condition.

For the subscale ‘Application’, the analysis demonstrated no significant effect of time, *F* (1,39) = .11, p = .74 or condition, *F*(1,39) = .19, p = .67. However, a significant interaction effect of time and condition was found, *F* (1,39) = 9.33, p < 0.01, η_p_^2^ = .85 (**Figure 4**). This finding suggests that the ACT-training had a significant favorable effect on the ‘Application’ subscale, compared to the control condition with an effect size of .193 (50, 51). The increase in ‘Application’ scores in the control condition was approximately as large as the decrease in the experimental condition (**Figure 5**).

Hurt-self: On the subscale no effect of time *F*(1,39) = 1.90, p = .18. or condition *F*(1,39) = 1.66, p = .21. was found. Furthermore, a non-significant trend interaction effect was found *F*(1,39) = 3.78, p = .056. suggesting a potential favorable effect in the intervention condition.

## Discussion

The focus of this study was to target self-stigmatization as it is known to be prevalent and detrimental for many patients in mental healthcare. To the best of our knowledge, the present study has been the first study to examine the impact of training MHCPs in ACT on reducing self-stigmatization among their patients. A significant effect of the intervention on the subscale Application was found; indicating a positive effect on reducing the internalization of mental health stereotypes among patients. Interestingly, scores on the Application subscale increased over time in the control condition. The hypothesis was not supported for the other subscales: Awareness, Agreement and Hurt-self (although favorable effect concerning Hurt-self were observed at a trend level). These findings suggest that the intervention did not result in significant changes in awareness of, agreement with, commonly held stereotypes, or self-esteem.

Self-stigmatization is commonly conceptualized as a process, encompassing identification with a stigmatized group and the internalization of negative attributes and stereotypes associated with that group (17). The subscales utilized in this study assess different stages of the self-stigmatization process and the subscales can only be interpreted separately. Awareness: this stage involves individuals becoming conscious of the existing stereotypes about mental illnesses, such as beliefs held by the public or mental healthcare professionals that attribute blame to individuals with mental illnesses for their problems (e.g., “The public/MHCP believes most people with mental illnesses are to blame for their problems.”). Agreement: After becoming aware of these stereotypes, individuals may then internalize and agree with them, reflecting their personal endorsement of such beliefs (e.g., “I think most people with mental illness are to blame for their problems.”). The first two stages of the process are not ‘personal’ yet; one can be aware of or agree with the statements without experiencing self-stigmatization. Application: At this stage, individuals apply the stereotypes onto themselves, attributing the negative stereotypes to their own experience with mental illness (e.g., “Because I have a mental illness, I am to blame for my problems.”). Hurt-self: As a consequence of the preceding stages, individuals may experience negative impacts on their self-esteem and self-worth, perceiving a loss of respect for themselves due to the belief that they are responsible for their problems (e.g., “I currently respect myself less because I am to blame for my problems.”). One implication of this model is that the ‘harmful’ effects of self-stigmatization do not occur until the later stages (Application and Hurt-self) when the person has internalized the stigma (32).

The subscale “Application” measures the internalization of negative stereotypes about mental health problems: from all the subscales this definition is most in line with the most commonly used definition of self-stigma (52) and was the only subscale that changed significantly. We propose that there was no significant effect found on the recognition of or the agreement with stereotypes alone (as measured by the Awareness and Agreement subscales) because these subscales measure beliefs that have been formed over a lifetime of socialization and were not affected by the ACT intervention. ACT does not attempt to decrease awareness or to change beliefs but to have more awareness of how these beliefs work and of how to create cognitive flexibility around them (for example to make people aware of the possibility not to apply the stereotypical beliefs they have onto themselves). So perhaps ACT doesn’t quickly modify the presence of self-stigmatizing psychological content, but alters its impact or function (e.g. believability). Varra et al. showed that ACT reduced the believability of barriers and greater psychological flexibility mediated the impact of the intervention in clinicians (53). Their results support the idea that acceptance-based interventions may be helpful in addressing the psychological factors related to poor adoption of evidence-based treatments. Unlike our study Hayes et al. observed that ACT also significantly changed the believability of stigmatizing attitudes (54).

Interestingly, the increase in Application scores in the control condition was approximately as large as the decrease in the experimental condition. A theory that could explain the observed increase of Application scores in the control condition can be found in the Ironic Process Theory. This theory states that negative (self)beliefs are partly suppressed into the subconscious (55). Addressing beliefs for instance through measurement with stereotypical statements has the potential to increase individuals’ awareness of them and hence can result in higher scores over time (56). An explanation for why this effect did not appear in the other subscales (Awareness and Agreement) is that the latter two scales measure ‘general’ beliefs, the subscale “Agreement” aims to measure the agreement with commonly held stereotypes about individuals with mental health problems and the subscale of Awareness measures the knowledge of common mental health stereotypes in society. Possibly these scores are not affected by the Ironic Process Theory because general beliefs are less likely to be suppressed (52).

On the subscale ‘Agreement’ patients in both conditions scored lower over time meaning that, because treatment as usual which both conditions received or because of other factors, they agreed less with the stereotypical beliefs as stated in the ISMI-SF. Interestingly, despite a decrease in ‘Agreement’ with the commonly held stereotypes observed in the control condition, there was still an increase in the application of these stereotypes onto oneself. This indicates that even though patients agreed less with stereotypes over time they still continued to apply them to themselves and internalized them. It could be that somehow the explicit beliefs (conscious beliefs) changed but the implicit (subconscious) beliefs did not which caused the application of the beliefs onto oneself to remain.

Furthermore, it is noteworthy that the intervention did not result in significant changes in the Hurt-self subscale. This subscale holds significance within the stigmatization process, as it has been found to be correlated with self-esteem, self-efficacy, empowerment, and hope (29). It is possible that these deeply rooted self-esteem beliefs are less amenable to change within the scope of a single workshop and session with mental healthcare professionals. Additional interventions or prolonged engagement may be required to stimulate change in this particular area.

### Limitations and strengths

Several limitations and strengths of this study should be mentioned. Firstly, a larger sample size of participating MHCPs and patients would have provided a better opportunity to observe significant changes in more domains of self-stigmatization and gain a more comprehensive understanding of the intervention’s effectiveness. Secondly; the MHCP was aware which one of their patients was included in the study, which may have implications for how the MHCP interacted with the patient: to prevent the potential bias in future studies it might be better to keep the MHCP blind for which of their patients is included. Thirdly no follow-up data were gathered to observe the effects over time and no active intervention was offered in the control condition, thereby making it difficult to conclude whether non-specific factors are responsible for the effects.

We consider it a strength of this study that we introduced and tested a new approach in addressing self-stigmatization by shifting the “responsibility” of diminishing (self-)-stigmatization from the patient to the MHCP. Moreover, the effect on the application subscale found in the analyses supports the added value of future research on the potential of this alternative approach to reduce self-stigmatization. Future research could benefit from first developing specific interventions focusing on the different elements in the process of self-stigmatization. Given the current findings with a relatively non intensive intervention, we consider further research on this alternative approach valuable. We recommend considering the following aspects in future research: to measure potential effects in several patients of each MHCP, to offer a longer intensive training intervention in the experimental condition to enhance the effect of the intervention, additional training/follow-up booster ACT and to include a larger sample.

## Data Availability

The raw data supporting the conclusions of this article will be made available by the authors, without undue reservation.

## References

[1] Frost BG Tirupati S Johnston S Turrell M Lewin TJ Sly KA . An Integrated Recovery-oriented Model (IRM) for mental health services: evolution and challenges. BMC Psychiatry. (2017) 17:22. doi: 10.1186/s12888-016-1164-3 28095811 PMC5240195

[2] Pincus HA Spaeth-Rublee B Sara G Goldner EM Prince PN Ramanuj P . A review of mental health recovery programs in selected industrialized countries. Int J Ment Health Syst. (2016) 10:73. doi: 10.1186/s13033-016-0104-4 27956939 PMC5131415

[3] Castelein S Timmerman M van der Gaag M Visser E . Clinical, societal and personal recovery in schizophrenia spectrum disorders across time: States and annual transitions. Br J Psychiatry. (2021) 219:401–8. doi: 10.1192/bjp.2021.48 35048855 PMC8529640

[4] Salzer MS Brusilovskiy E Townley G . National estimates of recovery-remission from serious mental illness. Psychiatr Serv. (2018) 69:523–8. doi: 10.1176/appi.ps.201700401 29385961

[5] Van Weeghel J van Zelst C Boertien D Hasson-Ohayon I . Conceptualizations, assessments, and implications of personal recovery in mental illness: A scoping review of systematic reviews and meta-analyses. Psychiatr Rehabil J. (2019) 42:169. doi: 10.1037/prj0000356 30843721

[6] Martinelli A Ruggeri M . An overview of mental health recovery-oriented practices: potentiality, challenges, prejudices, and misunderstandings. J Psychopathol. (2020) 26:147–54. doi: 10.36148/2284-0249-353

[7] van Boekel LC Brouwers EP van Weeghel J Garretsen HF . Stigma among health professionals towards patients with substance use disorders and its consequences for healthcare delivery: systematic review. Tijdschr Psychiatr. (2015) 57:489–97. doi: 10.1016/j.drugalcdep.2013.02.018 26189417

[8] Moser LL Bond GR . Practitioner attributes as predictors of restrictive practices in assertive community treatment. J Am Psychiatr Nurses Assoc. (2011) 17:80–9. doi: 10.1177/1078390310394360 21659298

[9] Horsfall J Cleary M Hunt GE . Stigma in mental health: clients and professionals. Issues Ment Health Nurs. (2010) 31:450–5. doi: 10.3109/01612840903537167 20521914

[10] Schulze B . Stigma and mental health professionals: a review of the evidence on an intricate relationship. Int Rev Psychiatry. (2007) 19:137–55. doi: 10.1080/09540260701278929 17464792

[11] Wahl O Aroesty-Cohen E . Attitudes of mental health professionals about mental illness: A review of the recent literature. J Community Psychol. (2010) 38:49–62. doi: 10.1002/jcop.20351 42587375

[12] Hinshaw SP Stier A . Stigma as related to mental disorders. Annu Rev Clin Psychol. (2008) 4:367–93. doi: 10.1146/annurev.clinpsy.4.022007.141245 17716044

[13] Stull LG McGrew JH Salyers MP Ashburn-Nardo L . Implicit and explicit stigma of mental illness: attitudes in an evidence-based practice. J Nerv Ment Dis. (2013) 201:1072–9. doi: 10.1097/nmd.0000000000000056 24284643 PMC4031039

[14] Strack F Deutsch R . Reflective and impulsive determinants of social behavior. Pers Soc Psychol Rev. (2004) 8:220–47. doi: 10.1207/s15327957pspr0803_1 15454347

[15] Asendorpf JB Banse R Mücke D . Double dissociation between implicit and explicit personality self-concept: the case of shy behavior. J Pers Soc Psychol. (2002) 83:380–93. doi: 10.1037/0022-3514.83.2.380 12150235

[16] Peter L-J Schindler S Sander C Schmidt S Muehlan H McLaren T . Continuum beliefs and mental illness stigma: a systematic review and meta-analysis of correlation and intervention studies. Psychol Med. (2021) 51:716–26. doi: 10.1017/S0033291721000854 33827725 PMC8108391

[17] Link BG Struening EL Neese-Todd S Asmussen S Phelan JC . Stigma as a barrier to recovery: The consequences of stigma for the self-esteem of people with mental illnesses. Psychiatr Serv. (2001) 52:1621–6. doi: 10.1176/appi.ps.52.12.1621 11726753

[18] Angermeyer MC van der Auwera S Carta MG Schomerus G . Public attitudes towards psychiatry and psychiatric treatment at the beginning of the 21st century: a systematic review and meta-analysis of population surveys. World Psychiatry. (2017) 16:50–61. doi: 10.1002/wps.20383 28127931 PMC5269489

[19] Clement S Schauman O Graham T Maggioni F Evans-Lacko S Bezborodovs N . What is the impact of mental health-related stigma on help-seeking? A systematic review of quantitative and qualitative studies. Psychol Med. (2015) 45:11–27. doi: 10.1017/S0033291714000129 24569086

[20] Corrigan PW Watson AC . Understanding the impact of stigma on people with mental illness. World Psychiatry. (2002) 1:16–20. 16946807 PMC1489832

[21] Corrigan PW Watson AC Heyrman ML Warpinski A Gracia G Slopen N . Structural stigma in state legislation. Psychiatr Serv. (2005) 56:557–63. doi: 10.1176/appi.ps.56.5.557 15872164

[22] Henderson C Evans-Lacko S Thornicroft G . Mental illness stigma, help seeking, and public health programs. Am J Public Health. (2013) 103:777–80. doi: 10.2105/ajph.2012.301056 23488489 PMC3698814

[23] Livingston JD Boyd JE . Correlates and consequences of internalized stigma for people living with mental illness: a systematic review and meta-analysis. Soc Sci Med. (2010) 71:2150–61. doi: 10.1016/j.socscimed.2010.09.030 21051128

[24] Mittal D Sullivan G Chekuri L Allee E Corrigan PW . Empirical studies of self-stigma reduction strategies: A critical review of the literature. Psychiatr Serv. (2012) 63:974–81. doi: 10.1176/appi.ps.201100459 22855130

[25] Corrigan PW Bink AB Schmidt A Jones N Rusch N . What is the impact of self-stigma? Loss of self-respect and the "why try" effect. J Ment Health. (2016) 25:10–5. doi: 10.3109/09638237.2015.1021902 26193430

[26] Link BG Phelan JC . Conceptualizing stigma. Annu Rev Sociology. (2001) 27:363–85. doi: 10.1146/annurev.soc.27.1.363 41139587

[27] Cavelti M Kvrgic S Beck EM Rüsch N Vauth R . Self-stigma and its relationship with insight, demoralization, and clinical outcome among people with schizophrenia spectrum disorders. Compr Psychiatry. (2012) 53:468–79. doi: 10.1016/j.comppsych.2011.08.001 21956043

[28] Kaushik A Kostaki E Kyriakopoulos M . The stigma of mental illness in children and adolescents: A systematic review. Psychiatry Res 243. (2016) 243:469–94. doi: 10.1016/j.psychres.2016.04.042 27517643

[29] Corrigan PW Druss BG Perlick DA . The impact of mental illness stigma on seeking and participating in mental health care. Psychol Sci Public Interest. (2014) 15:37–70. doi: 10.1177/1529100614531398 26171956

[30] Muntaner C Ng E Vanroelen C Christ S Eaton WW . Social stratification, social closure, and social class as determinants of mental health disparities. In: Handbook of the sociology of mental health, 2nd ed. Springer Science + Business Media, New York, NY, US (2013). p. 205–27.

[31] Wood L Byrne R Varese F Morrison AP . Psychosocial interventions for internalised stigma in people with a schizophrenia-spectrum diagnosis: A systematic narrative synthesis and meta-analysis. Schizophr Res. (2016) 176:291–303. doi: 10.1016/j.schres.2016.05.001 27256518

[32] Corrigan PW Morris SB Michaels PJ Rafacz JD Rüsch N . Challenging the public stigma of mental illness: a meta-analysis of outcome studies. Psychiatr Serv. (2012) 63:963–73. doi: 10.1176/appi.ps.201100529 23032675

[33] Na JJ Park JL Lkhagva T Mikami AY . The efficacy of interventions on cognitive, behavioral, and affective public stigma around mental illness: A systematic meta-analytic review. Stigma Health. (2022) 7:127–41. doi: 10.1037/sah0000372 27371692

[34] Morgan AJ Reavley NJ Ross A Too LS Jorm AF . Interventions to reduce stigma towards people with severe mental illness: Systematic review and meta-analysis. J Psychiatr Res. (2018) 103:120–33. doi: 10.1016/j.jpsychires.2018.05.017 29843003

[35] Tsang HW Ching SC Tang KH Lam HT Law PY Wan CN . Therapeutic intervention for internalized stigma of severe mental illness: A systematic review and meta-analysis. Schizophr Res. (2016) 173:45–53. doi: 10.1016/j.schres.2016.02.013 26969450

[36] Corrigan PW Bink AB Fokuo JK Schmidt A . The public stigma of mental illness means a difference between you and me. Psychiatry Res. (2015) 226:186–91. doi: 10.1016/j.psychres.2014.12.047 25660735

[37] Helmus K Schaars IK Wierenga H de Glint E van Os J . Decreasing stigmatization: reducing the discrepancy between "Us" and "Them". An intervention for mental health care professionals. Front Psychiatry. (2019) 10:243. doi: 10.3389/fpsyt.2019.00243 31214053 PMC6555228

[38] Sreeram A Cross WM Townsin L . Anti-stigma initiatives for mental health professionals—A systematic literature review. J Psychiatr Ment Health Nurs. (2022) 29:512–28. doi: 10.1111/jpm.12840 35500153

[39] Masuda A Price M Anderson PL Schmertz SK Calamaras MR . The role of psychological flexibility in mental health stigma and psychological distress for the stigmatizer. J Soc Clin Psychol. (2009) 28:1244–62. doi: 10.1521/jscp.2009.28.10.1244 37804533

[40] Weinstein JH Wilson KG Drake CE Kellum KK . A relational frame theory contribution to social categorization. Behav Soc Issues. (2008) 17:40–65. doi: 10.5210/bsi.v17i1.406

[41] Masuda A Hayes SC Fletcher LB Seignourel PJ Bunting K Herbst SA . Impact of acceptance and commitment therapy versus education on stigma toward people with psychological disorders. Behav Res Ther. (2007) 45:2764–72. doi: 10.1016/j.brat.2007.05.008 17643389

[42] Faul F Erdfelder E Lang AG Buchner A . G*Power 3: a flexible statistical power analysis program for the social, behavioral, and biomedical sciences. Behav Res Methods. (2007) 39:175–91. doi: 10.3758/bf03193146 17695343

[43] NIMH . (2023). Available online at: https://www.nimh.nih.gov/health/statistics/mental-illness (accessed October 2, 2023).

[44] Dindo L Van Liew JR Arch JJ . Acceptance and commitment therapy: a transdiagnostic behavioral intervention for mental health and medical conditions. Neurotherapeutics. (2017) 14:546–53. doi: 10.1007/s13311-017-0521-3 28271287 PMC5509623

[45] Hayes S Strosahl K Wilson K . Acceptance and Commitment Therapy: The Process and Practice of Mindful Change. New York City: The Guilford Press (2011).

[46] Masuda A Hayes SC Lillis J Bunting K Herbst SA Fletcher LB . The relation between psychological flexibility and mental health stigma in acceptance and commitment therapy: A preliminary process investigation. Behav Soc Issues. (2009) 18:25–40. doi: 10.5210/bsi.v18i1.2525

[47] Sheppard SC Forsyth JP Hickling EJ Bianchi J . A novel application of acceptance and commitment therapy for psychosocial problems associated with multiple sclerosis: results from a half-day workshop intervention. Int J MS Care. (2010) 12:200–6. doi: 10.7224/1537-2073-12.4.200

[48] Brohan E Slade M Clement S Thornicroft G . Experiences of mental illness stigma, prejudice and discrimination: a review of measures. BMC Health Serv Res. (2010) 10:80. doi: 10.1186/1472-6963-10-8 20338040 PMC2851715

[49] Rüsch N Lieb K Bohus M Corrigan PW . Self-stigma, empowerment, and perceived legitimacy of discrimination among women with mental illness. Psychiatr Serv. (2006) 57:399–402. doi: 10.1176/appi.ps.57.3.399 16525000

[50] Cohen J . Statistical Power Analysis for the Behavioral Sciences. 2nd ed. New York: Routledge (1988).

[51] Cohen J . Statistical power analysis for the behavioral sciences. New York: Routledge (2013).

[52] Vogel DL Wade NG Wester SR Larson L Hackler AH . Seeking help from a mental health professional: the influence of one's social network. J Clin Psychol. (2007) 63:233–45. doi: 10.1002/jclp.20345 17211874

[53] Varra AA Hayes SC Roget N Fisher G . A randomized control trial examining the effect of acceptance and commitment training on clinician willingness to use evidence-based pharmacotherapy. J Consult Clin Psychol. (2008) 76:449–58. doi: 10.1037/0022-006x.76.3.449 18540738

[54] Hayes SC Bissett R Roget N Padilla M Kohlenberg BS Fisher G . The impact of acceptance and commitment training and multicultural training on the stigmatizing attitudes and professional burnout of substance abuse counselors. Behav Ther. (2004) 35:821–35. doi: 10.1016/S0005-7894(04)80022-4

[55] Wegner DM . Ironic processes of mental control. psychol Rev. (1994) 101:34–52. doi: 10.1037/0033-295X.101.1.34 8121959

[56] Smart L Wegner DM . Covering up what can't be seen: Concealable stigma and mental control. J Pers Soc Psychol. (1999) 77:474–86. doi: 10.1037/0022-3514.77.3.474 10510504

