# Correction: Investigating the impact of acceptance and commitment therapy for mental healthcare professionals: the effect on patients´ self-stigmatization, a pilot study

**DOI:** 10.3389/fpsyt.2026.1894786

**Published:** 2026-08-24

**Authors:** Kim L. Helmus, Marilon van Doorn, Mariken B. de Koning, Inez Myin-Germeys, Frederike N. Schirmbeck, Therese A. M. J. van Amelsvoort, Dorien H. Nieman, Monique W. M. Jaspers, Arne Popma, Lieuwe de Haan

**Affiliations:** 1 Research Mental Health, Amsterdam Medical Centre, Amsterdam, Netherlands; 2 Neurology and Psychiatry Department, Amsterdam Universitair Medisch Centrum, Amsterdam, Netherlands; 3 Severe Mental Health Care Department (Mentrum), Arkin, Amsterdam, Netherlands; 4 Research Department, Arkin, Amsterdam, Netherlands; 5 Faculty of Medicine, Katholieke Universiteit Leuven, Kortrijk, Belgium; 6 Faculty Health, Medicine and Life Sciences, Maastricht University Medical Centre, Maastricht, Netherlands; 7 Department of Medical Informatics, Amsterdam Public Health Institute, Amsterdam, Netherlands

**Keywords:** self-stigmatization, acceptance and commitment therapy (act), mental healthcare, professionals, stigma & awareness

There was a mistake in **Figures 3**–**5** as published. The wrong images were associated with each figure. The corrected figures appears below.

**Figure 3:**
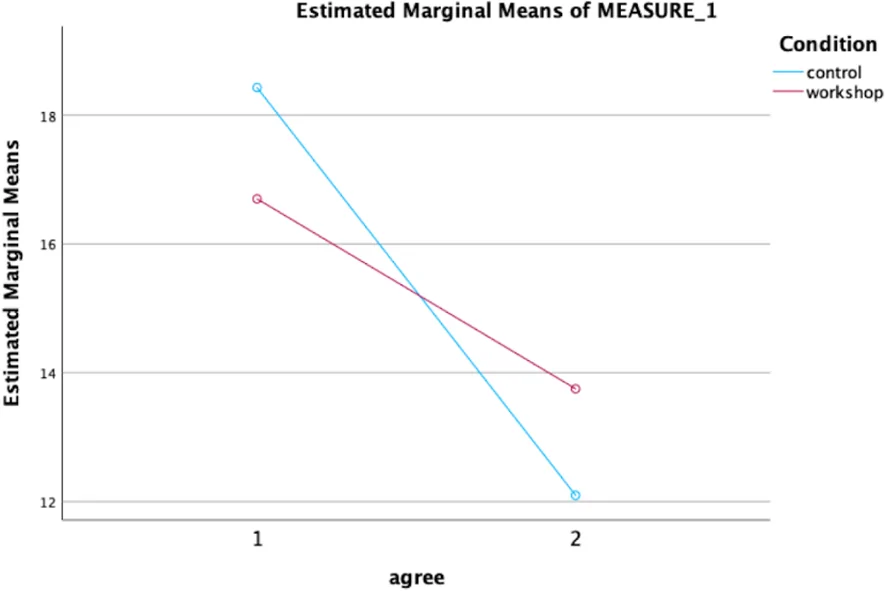
Pre- and post-test means (SD) of Agreement scores per condition.

**Figure 4:**
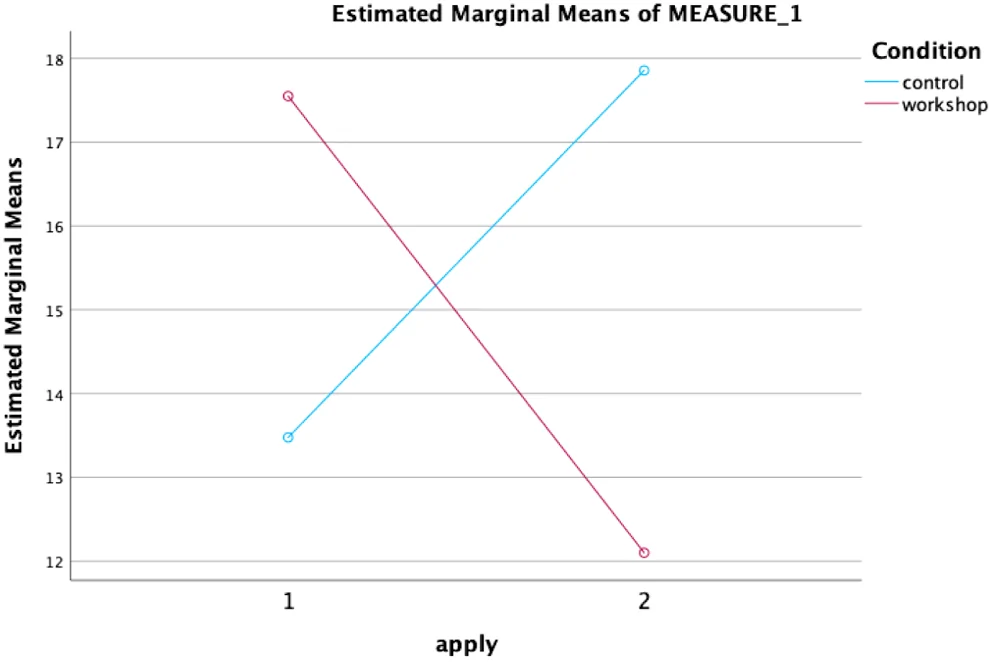
Pre- and post-test means (SD) of Application scores per condition.

**Figure 5:**
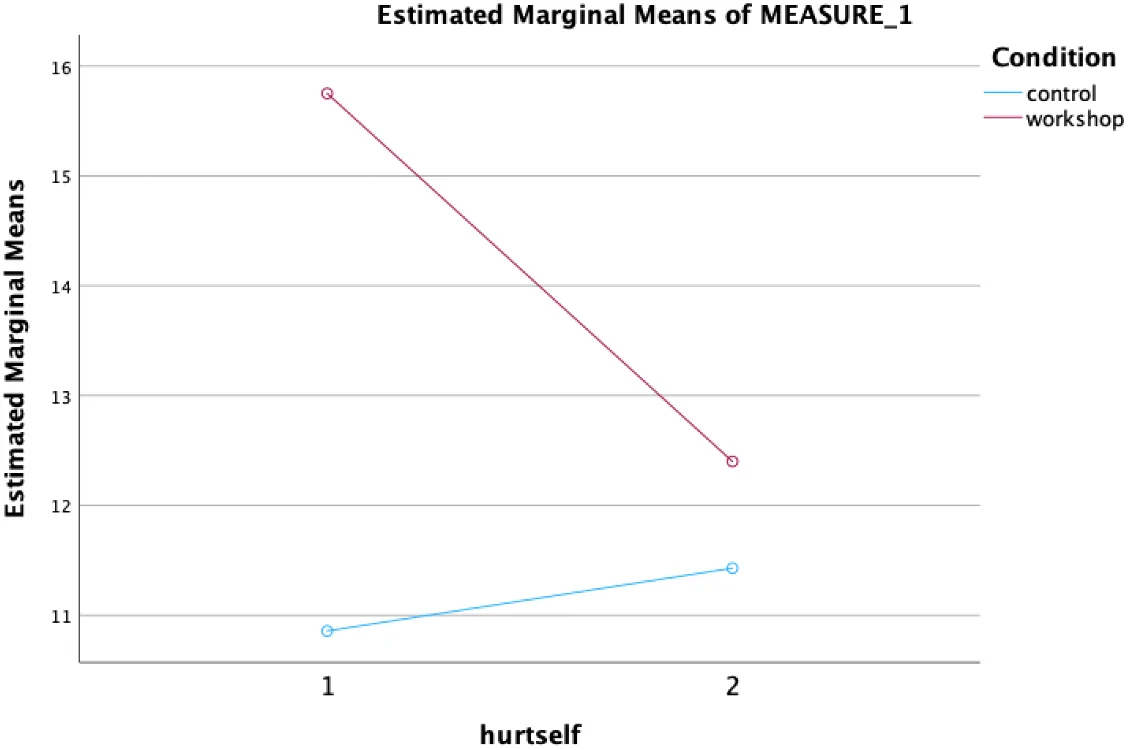
Pre- and post-test means (SD) of Hurt-self scores per condition.

